# Temporal Appearance of Enhanced Innate Anxiety in Alzheimer Model Mice

**DOI:** 10.3390/biomedicines11020262

**Published:** 2023-01-18

**Authors:** Adrienn Szabó, Szidónia Farkas, Csilla Fazekas, Pedro Correia, Tiago Chaves, Eszter Sipos, Bernadett Makkai, Bibiána Török, Dóra Zelena

**Affiliations:** 1 Centre for Neuroscience, Szentágothai Research Centre, Institute of Physiology, Medical School, University of Pécs, 7624 Pécs, Hungary; 2 Laboratory of Behavioral and Stress Studies, Institute of Experimental Medicine, 1083 Budapest, Hungary; 3 János Szentágothai School of Neurosciences, Semmelweis University, 1085 Budapest, Hungary

**Keywords:** Alzheimer’s disorder, 3xTg-AD mice, anxiety, fox odor, open-field, amyloid beta, Tau protein

## Abstract

The prevalence of Alzheimer’s disorder (AD) is increasing worldwide, and the co-morbid anxiety is an important, albeit often neglected problem, which might appear early during disease development. Animal models can be used to study this question. Mice, as prey animals, show an innate defensive response against a predator odor, providing a valuable tool for anxiety research. Our aim was to test whether the triple-transgenic mice model of AD shows signs of innate anxiety, with specific focus on the temporal appearance of the symptoms. We compared 3xTg-AD mice bearing human mutations of amyloid precursor protein, presenilin 1, and tau with age-matched controls. First, separate age-groups (between 2 and 18 months) were tested for the avoidance of 2-methyl-2-thiazoline, a fox odor component. To test whether hypolocomotion is a general sign of innate anxiety, open-field behavior was subsequently followed monthly in both sexes. The 3xTg-AD mice showed more immobility, approached the fox odor container less often, and spent more time in the avoidance zone. This effect was detectable already in two-month-old animals irrespective of sex, not visible around six months of age, and was more pronounced in aged females than males. The 3xTg-AD animals moved generally less. They also spent less time in the center of the open-field, which was detectable mainly in females older than five months. In contrast to controls, the aged 3xTg-AD was not able to habituate to the arena during a 30-min observation period irrespective of their sex. Amyloid beta and phospho-Tau accumulated gradually in the hippocampus, amygdala, olfactory bulb, and piriform cortex. In conclusion, the early appearance of predator odor- and open space-induced innate anxiety detected already in two-month-old 3xTg-AD mice make this genetically predisposed strain a good model for testing anxiety both before the onset of AD-related symptoms as well as during the later phase. Synaptic dysfunction by protein deposits might contribute to these disturbances.

## 1. Introduction

The prevalence of Alzheimer’s disease (AD) is increasing world-wide due to the aging society. During the pandemic’s and post-COVID scenarios, the loss of human lives and the implementation of physical distance measures might have a greater impact on the elderly, as neuropsychiatric symptoms are quite prevalent in this population [1]. From one side, anxiety may be a prodrome for symptomatic AD [2,3], and from other side, stress [4] and, based upon an umbrella review, anxiety are considered to represent a risk factor for dementia [5]. There is a longitudinal change during the course of the disorder, e.g., anxiety being more severe in the early onset AD forms than in the late onset forms [6].

The triple transgenic Alzheimer’s disease strain (3xTg-AD) is a common mouse model used for studying the pathology and mechanism of AD [7,8]. Pathological hallmarks appear gradually, normally detectable from six months onwards [9]. Earlier studies examined the anxiety of this 3xTg-AD mice induced by bright, open spaces using open-field (OF), elevated plus maze (EPM), or light-dark box (LD) behavioral tests (see Supplementary Table S1).

Although OF was examined between 2 and 19 months of age, in most cases, only one age group was observed. Only one study compared four ages from 3 till 12 months [7]. Approximately one third of the studies used both sexes, while another third reported only males or females. Nevertheless, the results were equivocal, with some authors reporting no difference between age-matched controls and 3xTg-AD mice (approximately half of the studies), while others found significantly enhanced anxiety (half of the studies regarding EPM and LD; third of the OF tests). Controversially, even reduced rather than enhanced anxiety was also detected (third of the OF tests, and 1-1 study for EPM and LD). When freezing was reported, it was always elevated in 3xTg-AD compared to controls. Interestingly, even the same research group using EPM in six-month-old 3xTg-AD mice reported reduced [10], unchanged [11], as well as enhanced [12] anxiety. Thus, the question, whether the 3xTg-AD mice model reflects the expected enhanced anxiety remains to be elucidated. One possible explanation for the discrepancies might be the temporal appearance of the symptoms. Therefore, we conducted longitudinal studies covering a wide age-range.

Moreover, we were focusing on the highly conservative avoidance of predator odor, another anxiety modality not examined before. Predatory cues are universal danger signals in all vertebrate species, including humans, modulating behavioral responses on the approach-avoidance dimension (e.g., exploration vs hiding) with significant autonomic and stress axis activation [13]. Prey animals, such as mice or rats, show innate defensive responses upon exposure to predator odor, providing a valuable means for studying the neurobiology of anxiety [14]. A monomolecular component of fox odor (i.e., 2-methyl-2-thiazoline (2MT)) is commercially available and often used as negative emotional challenge [15] to study avoidance behavior and anxiety [13,16].

The main neuropathological features of the disease are the deposition of extracellular amyloid beta (Aβ) plaques and intracellular neurofibrillary tangles (from hyperphosphorylated Tau protein; pTau) [17], which were observed at several points in the olfactory system (olfactory epithelium, piriform cortex, and in the olfactory bulb (OB)) in a variety of transgenic mouse models [18,19,20]. In OB, the effect of deposits may change over time during the course of dementia, and their presence should be confirmed in our local colony as a possible background mechanism of any observed changes in fox odor avoidance. While initially an increase in synaptic transmission and electrical activity was detected in transgenic mice [20,21,22], but at later age a decrease in activity was observed [20,23]. Thus, based upon this transient, silent period in electrical activity at six months of age we might expect a similar a transition state even in the behavior. We can even assume that symptoms will be detectable before and after (corresponding to the increased and decreased electrical activity, respectively) but not during this period in six-month-old animals, the known timepoint of appearance of AD hallmarks [9].

Here, we intended to examine the temporal appearance of innate anxiety in response to a predator cue comparing 3xTg-AD mice with age-matched controls. We hypothesized an enhanced anxiety. Thus, the 3xTg-AD mice should avoid the fox odor (2MT) more than the control animals. Predator odor stimuli are significant stressors shaping behavioral reactivity in the long-term and therefore it can be used as a model of post-traumatic stress disorder [13]. To avoid confounding effects of previous testing, separate animals were used for each age-groups. AD [24] as well as anxiety [25] are more prevalent among females. However, most of the preclinical (as well as clinical) studies are performed on males. Therefore, at selected age-groups (i.e., before and after the presumable transient period), a sex comparison was also performed. The detected hypolocomotion can be the consequence of enhanced anxiety from the predator odor or can reflect a general innate anxiety. To test the idea that 3xTg-AD mice have enhanced innate anxiety, another model, the OF was used reflecting innate anxiety from bright, open spaces [26]. Although OF was studied in 3xTg-AD mice before (see Supplementary Table S1), mostly a single age or sex were examined. Moreover, the results were equivocal (see earlier). Thus, testing our local colony seemed to be reasonable.

## 2. Materials and Methods

### 2.1. Animals

Adult 3xTg-AD mice were compared to age-matched C57Bl/6J control mice (*n* = 211; Jackson Laboratory, USA) [7,8]. All animals were group-housed (3–4 mice/cage) in Plexiglass chambers at constant temperature (22 ± 1 °C) and humidity (40 ± 10%) under reverse circadian light-dark cycle (lights-off at 8:00 a.m., lights-on at 8:00 p.m.). All behavioral tests were performed during the first half of the active (dark) cycle (between 9:00 and 14:00). Regular laboratory chow (Sniff, Soest, Germany) and tap water were available ad libitum.

Experiments were carried out in accordance with the European Communities Council Directive recommendations for the care and use of laboratory animals (2010/63/EU) and were reviewed and approved by the Animal Welfare Committee of the Institute of Experimental Medicine.

### 2.2. Experimental Design

Experiment 1. Fox odor avoidance in separate age-groups using males (2- (*n* = 14–10/group), 4- (*n* = 18–17/group), 6- (*n* = 11/group), 8- (*n* = 8/group), 12- (*n* = 4–6/group), 15- (*n* = 10/group), 18-month-old (*n* = 9–10/group)) and females (2- (*n* = 5–10/group) and 15-month-old (*n* = 5–7/group)). To have a comprehensive picture, we aimed to examine the temporal resolution in 2-month “bins”, however, after 12-month, due to insufficient number of animals, we switched to 3-month “bins”. Our main goal was to reveal temporal differences. Therefore, sex differences were addressed only at two ages (2 and 15 months, i.e., before and after the presumable transient period).

Experiment 2. Longitudinal, repeated (between 2 and 11 months of age) exploration of open-field behavior in male and female mice (*n* = 8/group at the beginning). As spending 5 min in an open arena does not have long term behavioral consequences, the same animals were examined repeatedly. The animals were tested monthly in slightly different environment to avoid habituation to the arena. As hypolocomotion was found repeatedly during 5 min testing, the question arose whether it is an initial anxiety in a new environment (which will be released after a while) or more a sign of innate anxiety from open, bright spaces, which does not diminish over time. To test this hypothesis, at the termination of our experiment (in 11-month-old mice), we conducted a more prolonged observation (30 min).

Experiment 3. Immunohistochemical confirmation of Aβ accumulation and pTau appearance in 2- and 12-month-old animals (i.e., before and after the presumable transient period [9]; female, *n* = 3; 3xTg-AD) in the OB, motor and somatosensory cortex, hippocampus, and basolateral amygdala regions as relevant regions for cognitive impairment (cortex, hippocampus), emotions (amygdala), or smell loss (OB).

### 2.3. Behavioral Testing

#### 2.3.1. Predator Odor Test Using 2-methyl-thiazoline (2MT)

We assessed the avoidance response to an ecologically relevant aversive stimulus (i.e., predator odor) by means of a synthetic analogue of a fox anogenital product (2-methyl-2-thiazoline; 2MT; #M83406 Merck (Sigma-Aldrich, Darmstadt, Germany), in a transparent Plexiglass arena (43 × 27 × 19 cm) [13]. Testing was carried out in a fume hood with medium-light intensity (120 lux) in covered arenas to equalize odor exposure across subjects. During testing, 2MT (40 μL in 1 mL distilled water, 50 μL/animal) was presented on a filter paper placed in a plastic vial cap affixed to the corner. One eighth of the box containing 2MT was defined as approach zone, while the distant quarter as avoidance zone. At start, animals were placed in the corner opposing the odor zone and were left to freely explore the covered arena for 10 min. Filter papers were immediately removed at the end of the test, then the testing arena was cleaned with 20% ethanol, wiped dry, and left ventilated for an additional 2 min before the next test. The following parameters were analyzed by EthoVision XT 15 software (Noldus, Wageningen, the Netherlands): (1) locomotor/horizontal exploratory activity (total distance moved in cm); (2) the time spent in the approach zone (corrected by locomotion: (duration spent in the approach zone)/(distance travelled), s/m); (3) the number of entries into the approach zone; (4) the time spent in the avoidance zone (corrected by locomotion: (duration spent in the avoidance zone)/(distance travelled), s/m); (5) the number of entries into the avoidance zone; and (6) time spent with immobility (not moving; s). Male mice were used throughout. However, 2- and 15-month females were also additionally involved. In these 2- and 15-month age-groups, additional behavioral variables were quantified, namely hand-scored by an experimenter blind to treatment groups (Solomon Coder, version beta 19.08.02, Budapest, Hungary). These variables were the following (time spent with the given behavior expressed as % of 10 min observation period): (1) freezing: no apparent movement; (2) sniffing the odor container; (3) rearing: vertical movement.

#### 2.3.2. Open-Field Test

Exploratory activity and anxiety-like behavior without predator stimuli was assessed in an open-field arena under medium-light intensity (120 lux). The exact size (50 × 45 × 15 cm or 40 × 40 × 30 cm) as well as the color (white or black) and smell (ethanol or soap) of the arena was changed from month to month to avoid loss of interest. Nevertheless, the plastic walls were always cleaned between animals and 4 animals were tested at a time. Mice were placed in the center and were allowed to explore the arena for 5 min [27]. At 11 months of age (last occasion), the 30-min test was conducted. The distance travelled was considered as a main parameter of locomotion. The inner 70% zone was considered as center, and time spent here was an index of anxiety. Here we did not calculate corrected duration spent in the center, while the animals were placed there and not moving animals stayed there, leading to a misleading reduced anxiety parameter. All behavioral variables were quantified using EthoVision XT 15 software.

### 2.4. Immunohistochemistry

Mice were anesthetized with a ketamine-xylazine solution (16.6 mg/mL and 0.6 mg/mL, respectively) and transcardially perfused with ice-cold phosphate-buffered saline (PBS), followed by ice-cold paraformaldehyde (4% PFA, VWR International, Leuven, Belgium, #28794.295; in TRIS VWR International, Leuven, Belgium, #103154M). Brains were rapidly removed and post-fixed overnight in 4% PFA at 4 °C, then incubated in a solution containing 30% sucrose (D-(+) saccharose puriss, Lach-Ner, Neratovice, Czech Republic, #57-50-1) in TRIS before slicing. Thirty-μm coronal sections were collected on a sliding microtome and stored in a cryoprotectant solution (containing 20% glycerine, Molar Chemicals, Halásztelek, Hungary, #03490-101-340; 30% ethylene glycol, Molar Chemicals, Halásztelek, Hungary, #03010-203-340) at −20 °C until immunohistochemical staining.

To investigate the amyloid plaques and hyperphosphorylated tau, we used peroxidase-based immunohistochemistry, with nickel-diaminobenzidine tetrahydrochloride (Ni-DAB; 3,3’-Diaminobenzidine tetrahydro-chloride hydrate, Merck (Sigma-Aldrich), Darmstadt, Germany, #D5637-1G; nickel(II) sulfate hexahydrate, Merck (Sigma-Aldrich, Darmstadt, Germany,#227676-500G ) visualization. Before staining for amyloid, a 95%, 10 min formic acid (Merck, Sigma- Aldrich, St. Louis, MO, USA, #F0507) pre-treatment was performed. Slices were incubated for 72 h, at 4°C in primary antibodies: anti-Aβ_1–42_ (1:500, polyclonal anti-rabbit, Invitrogen, Waltham, MA USA, #71–5800) and anti-phospho-Tau (1:500, monoclonal anti-mouse AT8, Invitrogen, Waltham, MA USA, #MN1020). As secondary, biotinylated anti-rabbit (1:200, Jackson ImmunoResearch, West Grove, PA, #111–065-003) and anti-mouse (1:200, Jackson ImmunoResearch, West Grove, PA, #715–065-151) antibodies were used, followed by an avidin-biotin treatment (VECTASTAIN Elite ABC-Peroxidase Kits, Vector Laboratories, Newark, CA, USA, #PK-6100) at room temperature for 2 h. Visualization was performed with a Ni-DAB and glucose oxidase (Merck, Sigma-Aldrich, St. Louis, MO USA, #G7141-10KU) solution. The labelling was imaged using Nikon Eclipse Ei microscope with a Digital Sight 1000 camera at 4× magnification and representative images are presented.

### 2.5. Statistics

Data are expressed as mean ± standard error of the mean (SEM). Differences between groups were analyzed using StatSoft 13.5 (Tibco, Palo Alto, CA, USA) by mix or factorial ANOVA (factors: genotype and sex and repeated measure on age/time) followed by Fisher LSD post hoc analyses. Multiple regression analysis was used to reveal body weight influence. The significance level was set at *p* < 0.05 throughout. The details of the statistics are given in the Section 3.

## 3. Results

### 3.1. Fox Odor Avoidance Behavior

**Figure 1:**
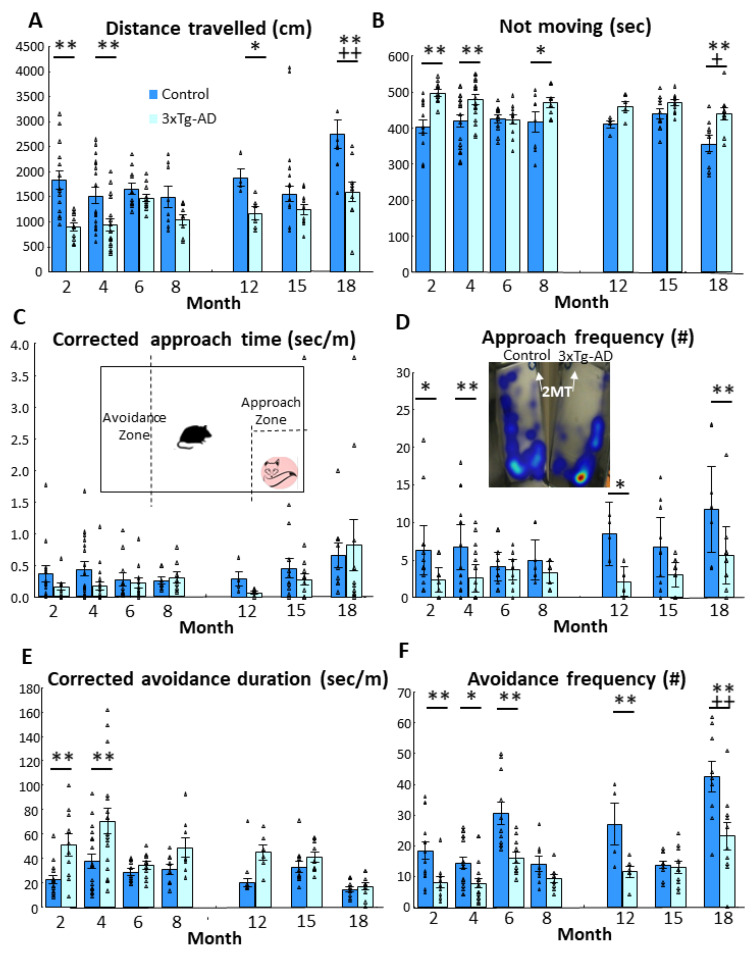
Fox odor avoidance behavior in male mice in different age groups (2, 4, 6, 8, 12, 15 and 18 months). (**A**) Distance travelled in centimeters during 10 min. The 3xTg-AD animals moved significantly less than the control animals (*p* < 0.01). Additionally, the control groups moved more with age (age: *p* < 0.01, genotype × age: *p* = 0.05). (**B**) Time spent without movement (“freezing”). Consistently with the previous result, the 3xTg-AD animals spent more time immobile (*p* < 0.01). In both groups, the tendency to freeze decayed with age (*p* < 0.01). (**C**) Corrected approach time to fox odor zone and representative image of the fox odor test. Due to differences in locomotion between the genotypes we opted to measure the approach time in s/m. In both groups, older animals spent more time near the fox odor (2-methyl-2-thiazoline, 2MT; *p* < 0.05). (**D**) Frequency of approaching the odor zone and illustrative example of mouse activity by heatmap (the warm (i.e., red) areas’ values are high and the cold (i.e., dark blue) areas’ values are low). The 3xTg-AD approached the 2MT containing zone less frequently (*p* < 0.01), however, this also increased with age (*p* < 0.05). (**E**) Locomotion corrected time spent in the avoidance zone. The 3xTg-AD animals spent more time in the avoidance zone than their age matched controls (*p* < 0.01). (**F**) Number of entering into the avoidance zone. As 3xTg-AD animals spent more time in the avoidance zone, they entered this zone less frequently (*p* < 0.01). Moreover, the age increased the number of entries in the control group, but not in the 3xTg-AD animals (*p* < 0.05). Data are shown as the mean ± SEM. The individual raw data points are represented as triangles. * *p* < 0.05, ** *p* < 0.01 vs. Control; + *p* < 0.05, ++ *p* < 0.01 vs. 2 m. # = amount of approach and/or avoidance frequency.

The 3xTg-AD animals moved significantly less than their age-matched controls (*p* < 0.01, Figure 1A, Table 1). Interestingly, the controls, but not the 3xTg-AD mice moved more with age (age: *p* < 0.01, genotype × age: *p* = 0.05). During the post-hoc test the genotype difference was not detectable in six-month-old mice, and in 8- and 15-month-old mice the difference was only marginally significant (0.05 < *p* < 0.10), with significant differences in other age-groups (at least *p* < 0.05). Coherent with the less movement, the 3xTg-AD animals spent more time in immobile, not moving position (*p* < 0.01, Figure 1B). The animals tended to freeze less with age (*p* < 0.01). However, once again, the six-month-old age group showed less difference between genotypes, with the two groups being almost identical (*p* = 0.966).

All animals were afraid of the fox odor, as reflected by 200–500-times more time spent in the avoidance than in approach zone despite only a double multiplier in their size. In fact, the 3xTg-AD mice in general avoided the 2MT smell more than their age matched controls (496.901 ± 48.829 vs. 279.099 ± 43.417 times difference) (F(1176) = 10.898, *p* < 0.01).

Due to differences in locomotion, we corrected the time spent in different compartments with it (s/m) (Figure 1C; Table 1). After correction, the previously significant genotype difference in approach time became non-significant. However, the age effect remained detectable (*p* < 0.05). Namely, with increasing age the animals spent significantly more time near the 2MT container than the younger animals. As expected, the approach frequency was lower in 3xTg-AD mice (*p* < 0.01), but this also increased with age (*p* < 0.05, Figure 1D). Although one might think that the avoidance time is a pure reverse of the approach time, there is a middle zone in between them (Figure 1C insert). Thus, avoidance requires activity. Indeed, 3xTg-AD animals were more afraid of the 2MT smell, spending more time in the avoidance zone than their age-matched controls, and this effect became highly significant after correction (*p* < 0.01, Table 1, Figure 1E). Interestingly, a longer amount of time was accompanied by fewer entries of 3xTg-AD mice into this avoidance zone (*p* < 0.01, Figure 1F). The number of entries increased with age in control, but not in 3xTg-AD animals (*p* < 0.05).

**Figure 2:**
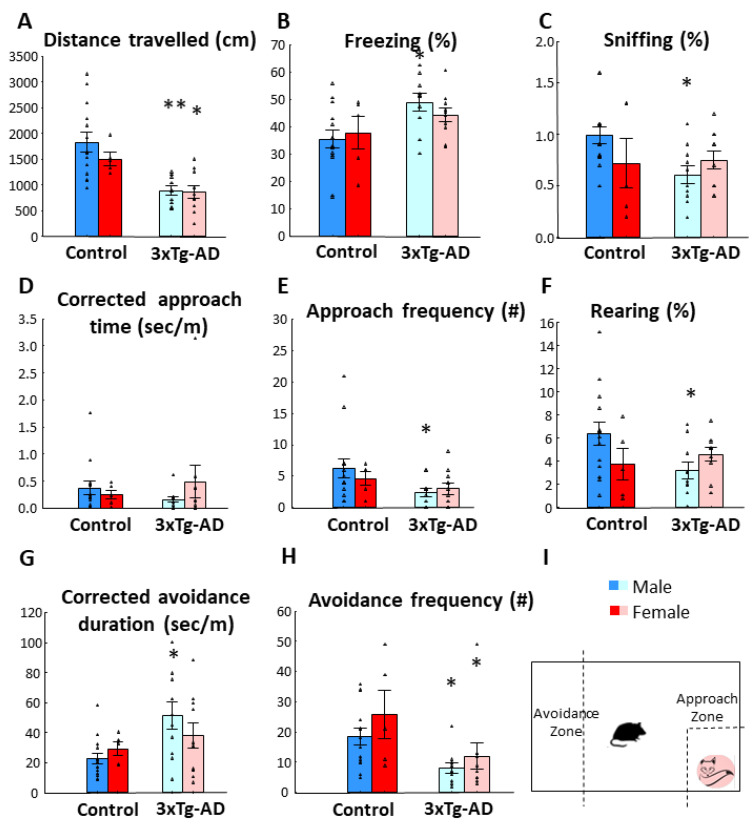
Fox odor avoidance behavior in 2-month-old male and female animals. **(A**) Distance traveled in centimeters during 10 min. The 3xTg-AD moved significantly less than the controls (*p* < 0.01) without sex difference. (**B**) Time spent freezing in percentage of 10 min observation period. The 3xTg-AD animals displayed increased freezing time (*p* < 0.01). (**C**) Time spent sniffing on the fox odor container. No statistical significance was observed in genotype or sex. (**D**) Corrected approach time to fox odor zone. No statistical significance was observed in genotype or sex. (**E**) Frequency in which the animals approached the odor zone. The 3xTg-AD group displayed lower frequency than the control group (*p* < 0.05). (**F**) Time spent rearing in percentage of 10 min observation period. No statistical significance was observed in genotype or sex. (**G**) Corrected avoidance duration. The 3xTg-AD animals spent more time in the avoidance zone than their age matched controls (*p* < 0.01). (**H**) Number of entries into the avoidance zone. As 3xTg-AD animals spent more time in the avoidance zone, they entered less often (*p* < 0.01). No main sex effect or genotype x sex interaction were observed in any of the presented parameters. (**I**) Schematic representation of the fox odor test. Data are shown as the mean ± SEM. The individual raw data points are represented as triangles. * *p* < 0.05, ** *p* < 0.01 vs. Control. # = amount of approach and/or avoidance frequency.

Similar to previous tests, the two-month-old 3xTg-AD animals moved significantly less (Figure 2A; Table 2.; *p* < 0.01). The tendency for lower approach frequency (*p* = 0.05), as well as significant genotype difference in corrected avoidance duration (*p* = 0.01), avoidance frequency (*p* < 0.01), as well as freezing time (*p* < 0.01) and frequency (*p* = 0.05), and frequency of sniffing the 2MT container (*p* < 0.05) all suggested enhanced anxiety of 3xTg-AD animals (Figure 2B–H). In two-month-old animals, there was no difference between the two sexes, except in freezing frequencies, which was higher in females without genotype influence (Male-Control: 45.786 ± 2.118; Male-3xTg-AD: 41.600 ± 2.344; Female-Control: 64.200 ± 6.102; Female-3xTg-AD: 52.800 ± 5.083).

**Figure 3:**
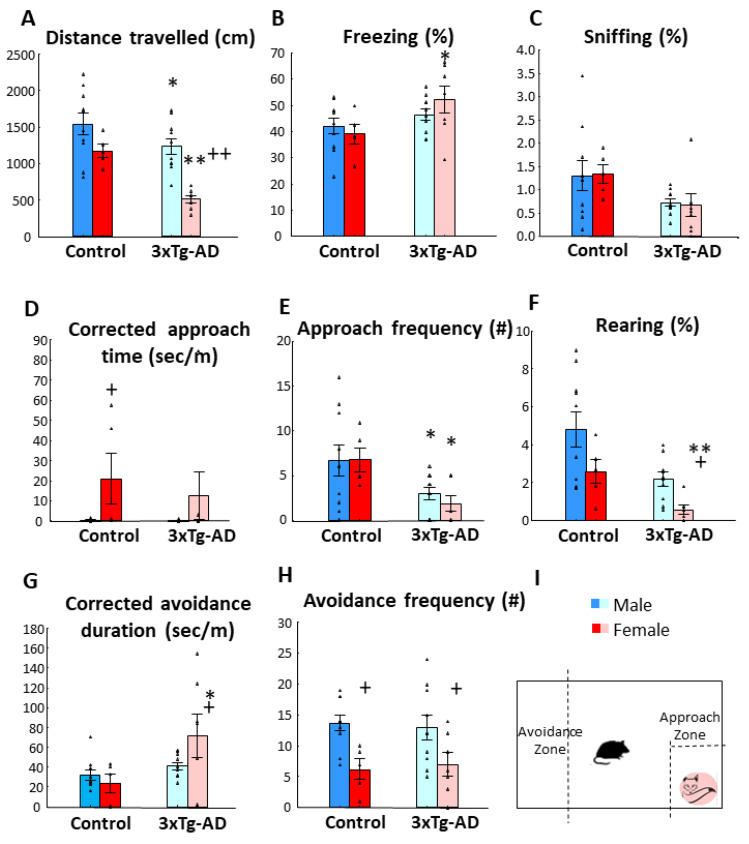
Fox odor avoidance behavior in 15-month-old male and female animals. (**A**) Distance traveled in centimeters during 10 min. The 3xTg-AD moved significantly less than the control group (*p* < 0.01). Additionally, the female moved less (*p* < 0.01) compared to males irrespective from their genotype. (**B**) Time spent freezing in percentage of 10 min observation period. The 3xTg-AD animals displayed increased freezing (*p* < 0.05). (**C**) Time spent sniffing on the fox odor container. The 3xTg-AD animals spent less time sniffing (*p* < 0.01) without sex difference. (**D**) Corrected approach time to fox odor zone. The female animals investigated the approached zone longer than males (*p* < 0.05). (**E**) Number of approaches to the odor zone. The 3xTg-AD group displayed lower frequency than the control group (*p* < 0.05). (**F**) Rearing activity. The 3xTg-AD reared less than controls (*p* < 0.05). (**G**) Corrected time spent in the avoidance zone. The 3xTg-AD animals spent more time in the avoidance zone than their age matched controls (*p* = 0.01). (**H**) Number of entries into the avoidance zone. The female animals entered into the avoidance zone less often (*p* = 0.01). No significant genotype x sex interaction was observed in any of the investigated parameters. (**I**) Schematic representation of the fox odor test. Data are shown as the mean ± SEM. The individual raw data points are represented as triangles. * *p* < 0.05, ** *p* < 0.01 vs. Control; + *p* < 0.05, ++ *p* < 0.01 vs. Male. # = amount of approach and/or avoidance frequency.

In 15-month-old animals, in accordance with previous results, the 3xTg-AD animals moved less both horizontally and vertically (*p* < 0.01). They also approach the fox odor container less (*p* < 0.05, Figure 3E) and spent more time far from it (*p* < 0.05, Figure 3G). Moreover, the 3xTg-AD animals sniffed the 2MT container less (*p* < 0.01) and for shorter periods (*p* < 0.05, Figure 3C) and spent more time freezing (*p* < 0.05, Figure 3B) than their controls. At this age, the sex difference was more pronounced. The females moved less (both horizontally (*p* < 0.01, Figure 3A) and vertically (*p* < 0.05, Figure 3F)) and spent more time near the 2MT container compared to males (*p* < 0.05, Figure 3D), irrespective of their genotype (Table 3). However, during post-hoc comparisons, 3xTg-AD females showed more significant changes than respective males.

### 3.2. Open-Field Behavior

**Figure 4:**
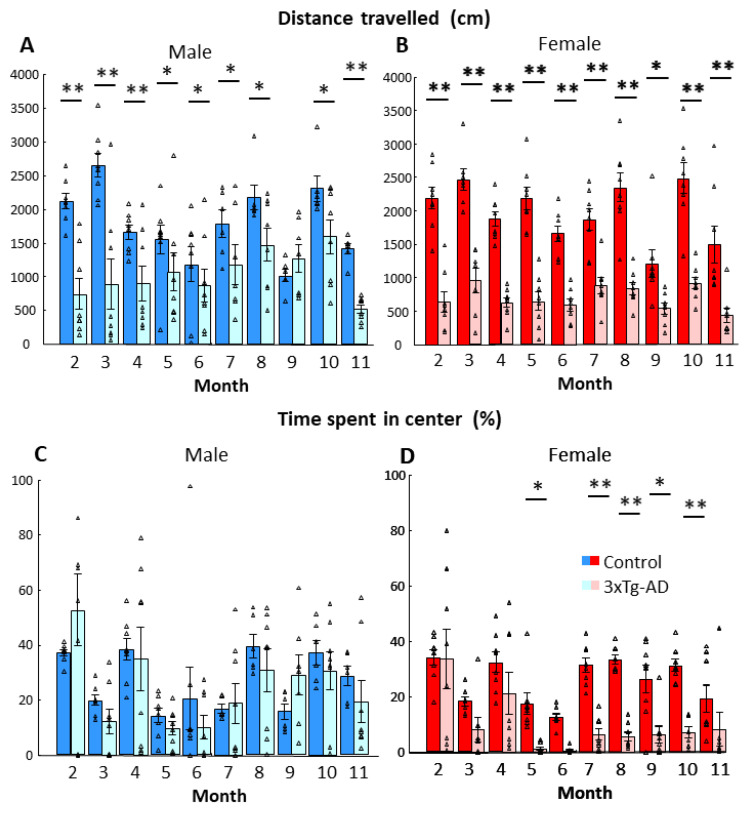
Open-field behavior during monthly testing between 2- and 11-month. (**A**,**B**) Distance traveled in centimeters during 5 min observation period. There was no overall difference between sexes, however the 3xTg-AD mice moved significantly less than age-matched controls (*p* < 0.01). (**C**,**D**) Percent of time spent in the center of the open field during 5 min observation. The 3xTg-AD animals spent less time in the center of the open field than the control group (*p* = 0.026). Data are shown as the mean ± SEM. The individual raw data points are represented as triangles. * *p* < 0.05, ** *p* < 0.01 vs. Control. The blues represent male (normal blue: control group, pale blue: intervention group), and the reds represent females ( normal red: control, pale red: intervention).

During repeated testing, the 3xTg-AD animals generally moved less (*p* < 0.01, Figure 4A,B; Table 4). Although there was no overall difference between sexes, there was a tendency with more pronounced difference between genotypes in females than in males (*p* = 0.07). In females, all post-hoc comparisons between genotypes were significant, while nine-month-old males did not show genotype difference.

The time spent in the center of the arena was generally less in 3xTg-AD than in control animals (*p* = 0.026, Figure 4C,D; Table 4). However, females spent less time in the center than males (*p* = 0.011), with a more pronounced difference between genotypes in females than males (*p* = 0.049). In fact, post-hoc testing found genotype differences only in females.

Despite no clear correlation between body weight and emotionality [28], we cannot rule out the possibility that changes in body weight influenced the above-mentioned parameters. In our hands, the initial body weight at two months was higher, as expected, in males than in females (Male-Control: 24.300 ± 0.705 g; Male-3xTg-AD: 21.563 ± 0.457 g; Female-Control: 19.463 ± 0.227 g; Female-3xTg-AD: 19.100 ± 0.293 g). The body weight increase during the 10-month observation period was higher in female 3xTg-AD group compared to all other groups (Male-Control: 43.860 ± 4.765%; Male-3xTg-AD: 39.628 ± 3.002%; Female-Control: 33.091 ± 1.497%; Female-3xTg-AD: 57.188 ± 5.954%; Table 4). Multiple regression analysis did not reveal any significant influence of body weight on locomotion. However, 7–9-month old 3xTg-AD females might spend less time in the centrum parallel with their higher body weight (F_(10,16)_ = 5.485, *p* < 0.01).

**Figure 5:**
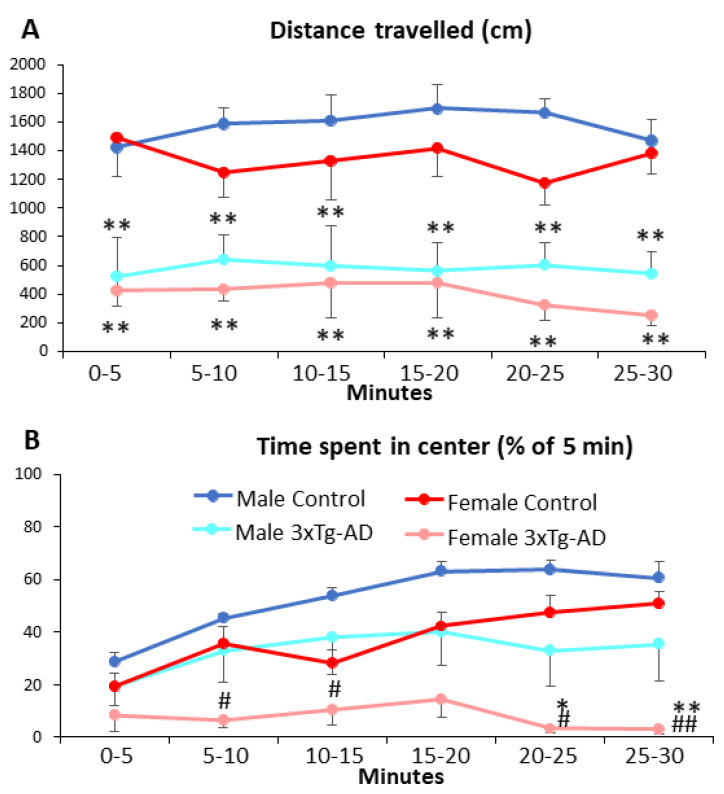
Open-field behavior during a 30 min observation period in 11-month-old animals. (**A**) Distance traveled in centimeters across 5 min time bins. The 3xTg-AD animals moved significantly less as their respective controls during the whole 30 min (*p* < 0.01). There was no difference between sexes. (**B**) Percent of time spent in the center in 5 min time bins. The control animals spent more and more time in the center of the arena, suggesting a reduced anxiety over time (*p* < 0.01), whereas the 3xTg-AD spent significantly less time in the center of the arena (*p* < 0.01) without habituation over time (genotype × time: *p* < 0.01). Data are shown as the mean ± SEM. * *p* < 0.05, ** *p* < 0.01 vs. respective control; # *p* < 0.05, ## *p* < 0.01 vs. respective male.

In 11-month-old animals, the 3xTgAD animals moved significantly less during the whole 30 min observation period (*p* < 0.01, Figure 5A, Table 5). The sex as well as the time had no influence on this parameter (i.e., no habituation was observed). Regarding the time spent in the center (Figure 5B), the control animals spent more and more time in the center as a sign of reduced anxiety over time (time as well as genotype × time: *p* < 0.01), while the 3xTg-AD animals spent significantly less time in the center (*p* < 0.01). Females were more anxious, spending less time in the central compartment (*p* = 0.01).

### 3.3. Immunohistochemical Confirmation of Temporal Appearance of the Histological Hallmarks

**Figure 6:**
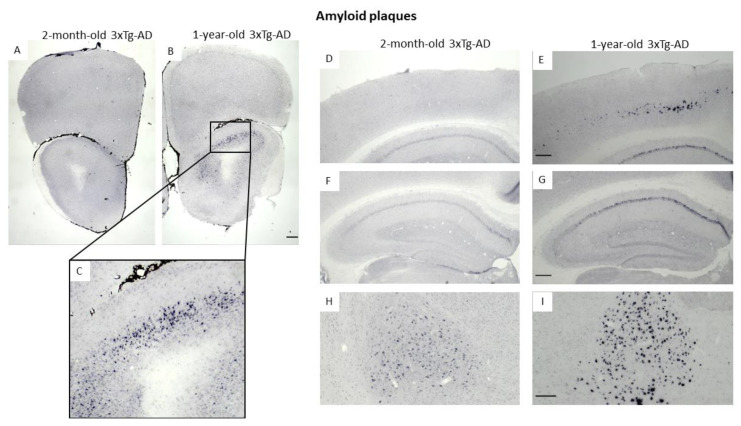
Amyloid-β_1–42_ plaques stained with Ni-DAB immunohistochemistry in 2-month-old and 1-year-old 3xTg-AD mice. The images show different brain areas: the olfactory bulb (**A**–**C**), motor- and somatosensory-cortex, (**D**,**E**), hippocampus (**F**,**G**), basolateral and basomedial amygdala (**H**,**I**). Scale bar in (**B**,**E**,**G**) is 200 μm, and in (**I**) 100 μm.

**Figure 7:**
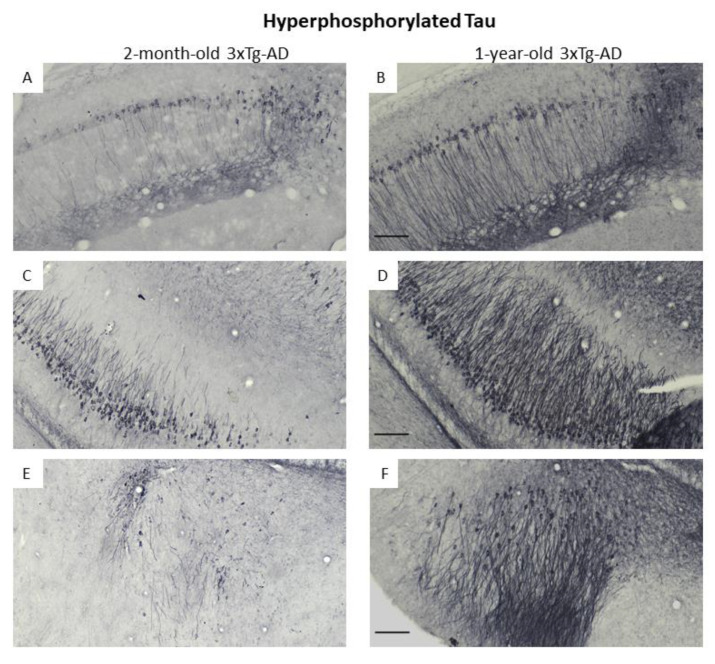
Phospho-Tau tangles stained with Ni-DAB immunohistochemistry in 2-month-old and 1-year-old 3xTg-AD mice. The images show different brain areas, like: pyramidal cell layer in the CA1 region of the hippocampus (**A**,**B**), pyramidal cell layer in the CA3 region of the hippocampus (**C**,**D**) and basolateral amygdaloid nucleus, amygdalopiriform area and entorhinal cortex (**E**,**F**). Scale bar is 100 μm.

Using Ni-DAB immunohistochemistry, we were able to confirm the progressive appearance of the classical hallmark of AD (Aβ and pTau) in brain areas important for memory formation (e.g., hippocampus; Figure 6F,G and Figure 7A–D) as well as in the amygdala, an important center of emotions including anxiety (Figure 6H,I and Figure 7E,F). Moreover, the deposits were detectable in the OB (Figure 6A–C) and piriform cortex (Figure 7E,F), brain areas participating in olfaction [29]. Interestingly, Aβ was only minimally present in two-month-old animals, while the signal intensity for pTau was already high in this age group. Nevertheless, the number of positively stained cells (both for Aβ as well as pTau) was higher in the brain of one-year-old animals than in two-month-old ones.

## 4. Discussion

Increased innate anxiety was detected in 3xTg-AD animals from two months of age, which is in line with human data indicating that anxiety may be a prodrome for symptomatic AD. Furthermore, an early (already at two months of age) appearance of pTau was also observed in emotionally relevant brain areas (i.e., amygdala, Figure 7E), which might contribute to the early synaptic disturbances [30,31], leading to anxiety-like symptoms. On the other hand, the early appearance of anxiety may enhance the development of AD pathology. Indeed, in 12-month-old animals, more deposits (both Aβ and pTau) were detectable than in two-month-old mice. These agglomerates may fatally destroy the synapsis, leading to a vicious circle, i.e., enhanced anxiety. However, this connection between pTau and anxiety needs further clarification.

In accordance with our hypothesis, around six months of age, there was a transient period with less pronounced anxiety-like behavior. This might be explained by an early increase in synaptic transmission of the 3xTg-AD mice [20,21,22] followed by a decrease in old age [20,23] leading to a transient remission of the symptoms between the two periods.

It is quite obvious that fox-odor influences the behavior of a mouse through the olfactory system, as the transient receptor potential ankyrin 1 knockout mice, lacking this receptor in their olfactory system, did not avoid the fox odor [29]. Human studies suggested that olfactory loss predicts the onset of subsequent dementia [32,33]. Furthermore, it may aggravate the symptoms leading itself to cognitive decline [34]. A similar loss of smell has been shown in several animal models of AD including 3xTg-AD [35,36]. However, six-month-old 3xTg-AD mice did not show olfactory deficit to social and non-social (almond and banana) odors [37]. The outcome of experiments evaluating olfaction by finding buried food [35,38] can be deeply confounded by metabolic/motivational processes, known to be altered in the 3xTg-AD mice [39]. In another case, sex-related olfactory function was measured, which can be influenced by sexual drive [36]. Only 1.5-year-old 3xTg-AD females, but not the one-year old ones, showed a deficit in discriminating strawberry from cinnamon [36]. Similarly, there was an age-related decline in finding the buried food only in females [35]. Interestingly, peanut butter was even more attractive to 3xTg-AD animals than to their control, while olfaction of peppermint, a repellent, was unaltered [40]. Nevertheless, in one-year-old 3xTg-AD animals PET-scan detected hypometabolism on brain areas relevant for olfaction (e.g., piriform cortex) [41]. Our immunohistochemical data confirmed the presence of Aβ (Figure 6A–C) and pTau (Figure 7E,F) in these areas (OB and piriform cortex). Moreover, in another model, the double transgenic AD mice, the Aβ plaque burden in the OB was detectable already at four months of age, but the behavioral alteration (less avoidance of the 2,4,5-trimethylthiazole (TMT) component of fox odor) was detectable in eight- but not in four-month-old mice [42]. All in all, although pathological morphological changes as well as altered olfactory behavior can be detected in aged 3xTg-AD mice, smell loss was not equivocal, and could hardly explain the enhanced avoidance of the predator odor (as in case of smell loss we could expect reduced avoidance) detected even in 18-month-old animals.

We repeatedly found reduced mobility of 3xTg-AD mice both during the fox odor, as well as in OF tests, independently from the sex. In a previous study, similarly to our results, the 3xTg-AD mice moved significantly less at 3, 9, and 12 months of age, but not at 6 months of age [7]. Further studies confirmed the lack of genotype difference in 6-month-old animals [4,43]. Again, a plausible explanation is the previously mentioned possible transient quiescence in synaptic dysfunction in this age-group. Another model of AD, the membrane protein seizure 6-like (SEZ6L) (a neuronal substrate of the AD protease BACE1) conditional knockout [44], displayed similarly decreased motor coordination. Although the loss of fine [45] (and even gross [46]) motoric might again be an early sign of AD, we speculated that in our hands the reduced locomotion was due to enhanced innate anxiety from bright, open spaces [26]. Enhanced novel environment-induced anxiety/freezing could be rule out, as during the 30-min observation period only the controls started to spend more time in the center, but not the 3xTg-AD mice, without temporal changes in locomotion in both genotypes (Figure 5). The drop of locomotion happens mostly during the first minute of a prolonged OF test [47], which can be explained by various factors, from new, surprising situations to an unknown experimenter. In our hands, the animals were tested repeatedly by the same experimenter. Thus, the initial high discomfort of the test might not have been present. Therefore, there was no drop in the distance travelled. Nevertheless, the genotype difference was present during the whole 30-min observation period, confirming the innate nature of anxiety induced by bright open spaces.

After the six-month transient period, the 3xTg-AD females were more afraid of both the predator odor and open spaces than males. In humans, females are generally more anxious [48], and their emotions highly influence their cognitive capability, too [49]. This female prevalence was also reflected in our older animals. We might assume that at younger age the sex hormonal system is immature, and therefore typical sex difference develops over time.

## 5. Conclusions

In summary, similarly to AD patients, the 3xTg-AD mice recapitulate some but not all (i.e., smell loss) behavioral and psychological symptoms of AD-like behaviors [50]. According to our present data, this mice strain might be a good model to examine the mechanism as well as possible treatment options of early onset anxiety in AD using the highly conservative avoidance of predator odor or considering anxiety caused by open spaces. Unlike cognitive tests, here, already two-month-old animals can be used, although the symptoms persist till at least 18 months.

## Figures and Tables

**Table 1:** Statistical data (results of factorial ANOVA) of fox odor avoidance behaviour (Experiment 1). The corresponding data are presented on Figure 1. Parameters were analyzed by EthoVision software. Male control and 3xTg-AD mice were compared in 2–18-month age range. Degree of freedom was 1132. Significant main effects are highlighted in red, while blue colour represents marginally significant difference.

Parameters	Genotype		Age		Genotype × Age	
	F	*p*	F	*p*	F	*p*
Distance	42.844	0.000	7.446	0.000	2.098	0.057
Not moving (s)	29.025	0.000	2.618	0.019	1.784	0.107
Approach duration	5.047	0.026	4.425	0.004	0.396	0.880
Corrected approach duration	1.431	0.233	2.598	0.020	0.559	0.762
Approach frequency	23.080	0.000	2.662	0.018	1.051	0.395
Avoidance duration	3.594	0.060	4.289	0.000	1.102	0.364
Corrected duration avoidance	19.306	0.000	6.812	0.000	1.750	0.114
Avoidance frequency	42.238	0.000	16.709	0.000	2.604	0.020

**Table 2:** Statistical data (results of factorial ANOVA) of fox odor test (Experiment 1). The corresponding data are presented on Figure 2. Parameters were analyzed by Solomon Coder by an experimenter blind to the treatment groups. Male and female control and 3xTg-AD mice age at 2-month. Degree of freedom was 1,35. Significant main effects are highlighted in red, while blue colour represents marginally significant difference.

Parameters	Genotype		Sex		Genotype x Sex	
	F	*p*	F	*p*	F	*p*
Distance	20.596	0.000	1.041	0.314	0.743	0.394
Not moving (s)	0.716	0.403	0.685	0.413	3.258	0.079
Approach duration	0.490	0.488	0.031	0.859	1.721	0.197
Corrected approach duration	0.004	0.945	0.271	0.605	1.267	0.267
Approach frequency	3.930	0.055	0.153	0.697	0.682	0.414
Avoidance duration	0.112	0.738	0.143	0.706	2.048	0.161
Corrected duration avoidance	6.061	0.018	0.178	0.675	1.707	0.199
Avoidance frequency	9.338	0.004	2.076	0.158	0.202	0.655
Freezing duration	7.464	0.009	0.090	0.765	0.866	0.358
Freezing frequency	4.116	0.050	14.862	0.000	0.882	0.354
Sniffing duration	2.004	0.165	0.354	0.555	3.181	0.083
Sniffing frequency	5.349	0.027	1.036	0.316	3.221	0.081
Rearing duration	1.342	0.254	0.414	0.523	3.971	0.054
Rearing frequency	3.408	0.073	0.324	0.573	2.704	0.109

**Table 3:** Statistical data (results of factorial ANOVA) of fox odor test (Experiment 1). The corresponding data are presented on Figure 3. Parameters were analyzed by Solomon Coder by an experimenter blind to the treatment groups. Male and female control and 3xTg-AD mice age at 15-month. Degree of freedom was 1,28. Significant main effects are highlighted in red, while blue colour represents marginally significant difference.

Parameters	Genotype		Sex		Genotype × Sex	
	F	*p*	F	*p*	F	*p*
Distance	14.351	0.000	18.199	0.000	1.890	0.180
Not moving (s)	17.604	0.000	17.633	0.000	3.181	0.085
Approach duration	2.119	0.156	7.446	0.010	1.833	0.186
Corrected approach duration	0.427	0.518	6.033	0.020	0.392	0.535
Approach frequency	10.092	0.036	0.146	0.704	0.208	0.651
Avoidance duration	0.547	0.465	4.197	0.049	0.034	0.853
Corrected duration avoidance	6.479	0.016	0.944	0.339	2.995	0.094
Avoidance frequency	0.000	0.978	13.149	0.001	0.162	0.690
Freezing duration	6.091	0.019	0.007	0.977	0.030	0.861
Freezing frequency	0.743	0.396	7.030	0.013	3.189	0.085
Sniffing duration	10.068	0.003	5.476	0.026	0.001	0.972
Sniffing frequency	9.042	0.006	0.489	0.490	0.292	0.593
Rearing duration	5.803	0.022	0.118	0.733	1.433	0.241
Rearing frequency	10.068	0.004	5.476	0.027	0.001	0.972

**Table 4:** Statistical data (results of mixed ANOVA) of open-field test (Experiment 2) and corresponding body weight in male and female, control and 3xTg-AD mice. The corresponding data are presented on Figure 4. Repeated measurements were conducted from 2 to 11 month of age. Significant main effects are highlighted in red, while blue colour represents marginally significant difference.

	Degree of Freedom	F	*p*
Distance travelled (cm)			
Genotype	1, 23	53.972	0.000
Sex	1, 23	1.017	0.324
Genotype × Sex	1, 23	3.567	0.072
Time	9, 207	20.267	0.000
Genotype × Time	9, 207	7.736	0.000
Sex × Time	9, 207	0.451	0.905
Genotype × Sex × Time	9, 207	2.822	0.004
Time spent in centrum (%)			
Genotype	1, 23	5.662	0.026
Sex	1, 23	7.561	0.011
Genotype × Sex	1, 23	4.317	0.049
Time	9, 207	13.458	0.000
Genotype × Time	9, 207	1.508	0.147
Sex × Time	9, 207	0.410	0.186
Genotype × Sex × Time	9, 207	1.382	0.198
Body weight (g)			
Genotype	1,26	1.554	0.224
Sex	1,26	57.509	0.000
Genotype × Sex	1,26	27.872	0.000
Time	9234	120.606	0.000
Genotype × Time	9234	0.694	0.714
Sex × Time	9234	1.429	0.176
Genotype × Sex × Time	9234	5.782	0.000

**Table 5:** Statistical data (results of mixed ANOVA) of open-field test (Experiment 2) in male and female, control and 3xTg-AD mice. The corresponding data are presented on Figure 5. Repeated measurements were conducted in 11-month-old animals during 30 min open-field test. Significant main effects are highlighted in red.

	Degree of Freedom	F	*p*
Distance travelled (cm)			
Genotype	1, 26	43.003	0.000
Sex	1, 26	1.940	0.175
Genotype × Sex	1, 26	0.030	0.865
Time	5, 130	0.963	0.443
Genotype × Time	5, 130	0.425	0.831
Sex × Time	5, 130	1.781	0.101
Genotype × Sex × Time	5, 130	1.031	0.402
Time spent in centrum (%)			
Genotype	1, 26	8.939	0.007
Sex	1, 26	7.856	0.010
Genotype × Sex	1, 26	0.572	0.457
Time	5, 130	11.730	0.000
Genotype × Time	5, 130	5.880	0.000
Sex × Time	5, 130	1.753	0.129
Genotype × Sex × Time	5, 130	1.610	0.163

## Data Availability

The data presented in this study are available on request from the corresponding author.

## Supplementary Materials

The following supporting information can be downloaded at: https://www.mdpi.com/article/10.3390/biomedicines11020262/s1, Table S1: Summary of the literature on 3xTg-AD and anxiety. Refs [51,52,53,54,55,56,57,58,59,60,61,62,63,64,65,66,67,68,69,70,71,72,73,74,75,76,77,78,79] are cited in the Supplementary Materials.
