# Supplementary material for: Temporal Appearance of Enhanced Innate Anxiety in Alzheimer Model Mice
Source: Biomedicines. 2023 Jan 18;11(2):262. doi: 10.3390/biomedicines11020262 (PMC9953677; doi:10.3390/biomedicines11020262)
Supplement: Supplementary file 1 [file biomedicines-11-00262-s001.zip › biomedicines-2095496-supplementary.pdf]

Supplementary Table S1. Summary of the literature on 3xTg-AD and anxiety (PubMed search) In the Reference the nationality represents the same research group / mice colony. For detailed analysis of the results see Introduction, third paragraph

Age	Sex	in 3xTg-AD to control	Reference
		Open field test	
		Parameter: time spent in the centrum	
2m	male	Ø	[51]
	female	↑	[51]
4m	male and female	Ø	[51]
2m, 6m	male and female	Ø	[52]
6m	male and female	Ø	[4]
	?	↓	[43] Chinese
7m	male	↑ (latency)	[53] Spanish 1
7-8m	female	↓ (icv cannula)	[54] Chinese
4- and 12-m	female	(↑) heterozygous	[55]
7.5-11m (range)	female	↑	[56]
8m	female	↓ (saline ip)	[57] Chinese
9m	?	↓ (icv cannula)	[58]
10-11m	male	Ø	[59]
11m	male	Ø	[60] Spanish 1
	?	Ø	[61]
	male	Ø	[53] Spanish 1
12m	male	Ø	[38] Spanish 1
13m	male	↑	[1] Spanish 1
14m	male and female	Ø	[62] Spanish 1
15m	male and female	Ø	[63] Spanish 1
18m	male?	(↑)	[64] Spanish 1
	female	Ø (latency)	[65] Spanish 1
19m	male and female	Ø (heterozygous)	[66]
2m	male and female	↑	[51]
4m	male and female	Ø	[51]
2m	male and female	↑	[51]
4m	male and female	Ø	[51]
4 and 7m	male and female	Ø	[67] Spanish 1
2m, 6m	male and female	↑	[52]
6m	male	↑	[4]
	female	Ø	[4]
7.5-11m (range)	female	↑	[56]
11m	male	Ø	[60] Spanish
10-11m	male	(↑)	[59]
12m	male	↑ (urine spots)	[68] Spanish 1
18m	female	Ø	[65] Spanish 1
		**Elevated plus maze test**	
		Parameter: time spent in the open arm	
2-4m	male and female	↓	[51]
6m	male and female	Ø	[4]
7.5-11m (range)	female	Ø	[56]
7-8m	female	↓	[54]

3-, 6-, 9m	female	↑	[7]
3-, 6-, 9-, 12m	male	Ø	[7]
6m	?	↓	[43] Chinese
8m	female	↓ (saline ip)	[57] Chinese
10-11m	male	Ø	[59]
11m	?	↓	[61] Chinese
12m	female	Ø	[7]
8-14m	female	Ø	[69]
Parameter: frequency of the entering the open arm			
6m	male	↑ (vehicle)	[70]
	female	Ø (vehicle)	[70]
Parameter: anxiety index (OA/OA+CA%)			
13m	male	↓	[71] Spanish 1
Parameter: time spent freezing			
6m	male and female	↑ (vehicle)	[70]
**Light-Dark box test**			
Parameter: time spent in the light area			
6m	?	↓	[43] Chinese
4 and 4m	male and female	Ø	[67] Spanish 1
7m	male	Ø	[53] Spanish 1
11m	male	↓	[53] Spanish 1
12m	male	Ø	[68] Spanish 1
	male/female	↓ (ip saline)	[72]
	male/female	↓	[73] Spanish 2
	male	Ø	[38] Spanish 1
14m	male and female	↑	[62] Spanish 1
15m	male	Ø (saline treated)	[74]
	female	↓	[75] Spanish 1
18m	male?	(↓)	[64] Spanish 1
	male/female (mixed)	Ø	[76]
Parameter: latency to enter the light area			
12m	male	↑	[77] Spanish 2
13m	male	↓	[71] Spanish 1
Parameter: time spent freezing			
12m	male	↑	[77] Spanish 2
**Marble burying** (perseverative and/or stereotyped-like behavior)			
Parameter: number of buried marbles			
12m	male	↑	[78] Spanish 1
12-16m, repeated	male vs female	↑	[79] Spanish 1
13m	male	Ø	[1] Spanish 1
14m	male and female	Ø	[62] Spanish 1
18m	male?	Ø	[64] Spanish 1
